# Systemic Inflammation and Complement Activation Parameters Predict Clinical Outcome of Severe SARS-CoV-2 Infections

**DOI:** 10.3390/v13122376

**Published:** 2021-11-26

**Authors:** Silke Huber, Mariam Massri, Marco Grasse, Verena Fleischer, Sára Kellnerová, Verena Harpf, Ludwig Knabl, Ludwig Knabl, Tatjana Heiner, Moritz Kummann, Magdalena Neurauter, Günter Rambach, Cornelia Speth, Reinhard Würzner

**Affiliations:** 1 Institute of Hygiene and Medical Microbiology, Medical University of Innsbruck, 6020 Innsbruck, Austria; silke.huber@i-med.ac.at (S.H.); mariam.massri@i-med.ac.at (M.M.); marco.grasse@i-med.ac.at (M.G.); verena.fleischer@i-med.ac.at (V.F.); sara.kellnerova@i-med.ac.at (S.K.); verena.harpf@i-med.ac.at (V.H.); magdalena.neurauter@i-med.ac.at (M.N.); guenter.rambach@i-med.ac.at (G.R.); cornelia.speth@i-med.ac.at (C.S.); 2 TyrolPath, 6511 Zams, Austria; ludwig.knabl@tyrolpath.at; 3 Department of Internal Medicine, Hospital St. Vinzenz, 6511 Zams, Austria; aon.964003992.knabl@aon.at; 4 Department of Anesthesia and Intensive Care Medicine, Hospital Reutte, 6600 Reutte, Austria; tatjana.heiner@bkh-reutte.at; 5 Department of Radiology, Medical University of Innsbruck, 6020 Innsbruck, Austria; m.kummann@i-med.ac.at

**Keywords:** viral infection, complement, SARS-CoV-2, infectious disease, NETosis, tracheal fluid

## Abstract

Overactivation of the complement system has been characterized in severe COVID-19 cases. Complement components are known to trigger NETosis via the coagulation cascade and have also been reported in human tracheobronchial epithelial cells. In this longitudinal study, we investigated systemic and local complement activation and NETosis in COVID-19 patients that underwent mechanical ventilation. Results confirmed significantly higher baseline levels of serum C5a (24.5 ± 39.0 ng/mL) and TCC (11.03 ± 8.52 µg/mL) in patients compared to healthy controls (*p* < 0.01 and *p* < 0.0001, respectively). Furthermore, systemic NETosis was significantly augmented in patients (5.87 (±3.71) × 10^6^ neutrophils/mL) compared to healthy controls (0.82 (±0.74) × 10^6^ neutrophils/mL) (*p* < 0.0001). In tracheal fluid, baseline TCC levels but not C5a and NETosis, were significantly higher in patients. Kinetic studies of systemic complement activation revealed markedly higher levels of TCC and CRP in nonsurvivors compared to survivors. In contrast, kinetic studies showed decreased local NETosis in tracheal fluid but comparable local complement activation in nonsurvivors compared to survivors. Systemic TCC and NETosis were significantly correlated with inflammation and coagulation markers. We propose that a ratio comprising systemic inflammation, complement activation, and chest X-ray score could be rendered as a predictive parameter of patient outcome in severe SARS-CoV-2 infections.

## 1. Introduction

Severe acute respiratory syndrome coronavirus 2 (SARS-CoV-2) belongs to the family of *Coronaviridae*, a large family of single-stranded RNA viruses that are pathogenic in humans and several animal species [1,2]. The SARS-CoV-2 infection, responsible for COVID-19 (coronavirus disease 2019), was described by mid-December 2019. It has spread globally ever since, and the outbreak was eventually declared a pandemic by mid-March 2020 [3]. Worldwide statistics have thus far reported 240 million confirmed infections and around five million deaths [4]. In Austria, the reported number of infected individuals has surpassed 775,000 cases [4,5]. Tyrol was the first Austrian federal state to be affected, with a number of well above 70,000 confirmed cases in the summer of 2021 [5]. Although the majority of presented infections are asymptomatic or are defined by mild respiratory symptoms, some infected individuals develop severe viral pneumonia, leading to acute respiratory distress syndrome (ARDS), ultimately resulting in the patient’s hospitalization and potential requirement of mechanical ventilation in the intensive care unit (ICU) [6,7].

Dysregulated complement activation appears to play an important role in the development of acute lung diseases induced by pathogenic viruses [8,9]. SARS-CoV-2 is the third strain of human pathogenic coronaviruses that resulted in an outbreak. Previous coronavirus strains, SARS-CoV-1 (China, 2002) and Middle East respiratory syndrome MERS-CoV (2012), are known to activate the complement system, consequently resulting in acute respiratory failure [10,11]. A recent study demonstrated the activation of the lectin pathway (LP) by SARS-CoV-2 via the binding of the LP recognition molecules MBL, FCN-2, and CL-11 to the viral spike and nucleocapsid proteins [12].

Complement is activated via three distinct pathways, thereby leading to the formation of highly potent immuno-active molecules, such as the anaphylatoxins C3a and C5a, as well as the terminal complement complex (TCC; sC5b-9)/membrane attack complex (MAC). C5a is a strong chemoattractant involved in the recruitment of inflammatory immune cells and the release of immuno-active molecules, which can instigate a “cytokine storm” [13]. Most importantly, C5a seems to play a major role in viral-induced acute lung injury [14]. Activated complement components are also known to interact with the coagulation cascade and induce NETosis, triggering neutrophils to release neutrophil extracellular traps (NETs) [15]. The contribution of complement activation to the progression of ARDS in SARS-CoV infections has been further confirmed in complement-deficient mice (C3^−/−^) [10]. This study elegantly demonstrated that the absence of complement significantly attenuated the severity of the respiratory disease despite the presence of a constant viral load. Furthermore, complement deposition has been reported in multiple organs of COVID-19 patients, including the lungs. Localization of C1q, C3, C4, and C5b-9 was observed in the capillaries of the interalveolar septa and on alveolar cells, highlighting the importance the complement system in inflammation and tissue damage [16].

Recently, SARS-CoV-2 infections, coupled with the development of acute respiratory syndrome, have been associated with an overreaction of the innate immune response, with complement playing a key role [17]. High levels of complement-activated products, C5a and sC5b-9, are observed in patients suffering from severe COVID-19 [18,19,20] and may serve as indicators of disease course severity [20]. A large retrospective observational study of more than 11,000 patients with suspected SARS-CoV-2 infections highlighted complement activation and coagulation as crucial risk factors of mortality and morbidity, independent of other prominent risk factors [21]. Additionally, a targeted genetic association study was deployed to identify specific single nucleotide polymorphisms (SNPs) in components of complement and coagulation pathways that are linked with the clinical outcome in infected individuals. The essential role that complement plays in the course of disease after a SARS-CoV-2 infection has prompted a closer look at complement proteins as potential therapeutic targets for COVID-19 [22]. For instance, a pilot study in severe COVID-19 patients unraveled the role of the anti-C5a monoclonal antibody, Soliris, as a potential therapeutic measure in COVID-related ARDS [23]. Despite the evident interest in unraveling the effect of increased systemic complement activation in SARS-CoV-2, only a few studies focus on complement activation in the lung and the kinetics of local and systemic complement activation under mechanical ventilation [17,24].

Here, we investigated the course of systemic and local complement activation in COVID-19 patients over the period of artificial respiration. Complement proteins have been previously found in the mucus of human tracheobronchial epithelial cells [25,26]. Patients under artificial respiration must be regularly relieved from tracheal secretions due to intubation. We therefore sought to acquire tracheal secretions from COVID-19 patients who underwent mechanical ventilation and analyze the amount of complement proteins and rate of complement activation over the period of artificial respiration. We intended to capture the molecular evidence of complement involvement in severe SARS-CoV-2 infections. In addition, we aimed herein to get insights into the kinetics of complement activation and to identify biomarkers for early prediction of mortality.

## 2. Materials and Methods

### 2.1. Patient Cohorts and Sample Collection

In this prospective longitudinal study, COVID-19 patients were recruited at the Department of Internal Medicine in the tertiary Hospital St. Vinzenz, Zams after PCR-confirmed or presumed SARS-CoV-2 infection following admission to the ICU between November 2020 and January 2021. Whole blood, collected in serum vials, and tracheal fluid from patients on mechanical ventilation were obtained every 24 h upon routine collection of samples for laboratory evaluation. Severely anemic patients were excluded from the study. Patients were classified as nonsurvivors upon death, during mechanical ventilation, or within an observational period of 14 days after extubation. The control group consisted of healthy individuals who were scheduled for surgery at the Department of Anesthesia and Intensive Care Medicine in Hospital Reutte within the same time period. Whole blood, collected in serum vials, and tracheal fluid from these healthy individuals was obtained during the surgery following a written consent. Samples collected from both hospitals were sent to our institute and processed within 24 h. Serum was obtained from whole blood samples by centrifugation (2000× *g*, 15 min, 22 °C). Tracheal fluid was analyzed for viral titer and secondary infections as described in Section 2.6 and Section 2.7 or supplemented with a protease inhibitor cocktail (Sigma-Aldrich, Merck KGaA, Darmstadt, Germany). Serum and supplemented tracheal fluid were stored in aliquots at −80 °C until further analysis.

### 2.2. Data Collection

Clinical data, including date of sampling, gender, age, mortality, and laboratory parameters, were acquired from the electronic medical record. Laboratory parameters included C-reactive protein (CRP; mg/L), procalcitonin (PCT; ng/mL), interleukin 6 (IL-6; pg/mL), white blood cell count (WBC; cells/µL), neutrophil count (cell/µL), platelet count (cell/µL), international normalized ratio (INR), and D-dimer levels (mg/L). Furthermore, results of thorax computer radiography (CR) were collected.

### 2.3. Chest X-ray Score Analysis

Chest X-rays (CXR) acquired from each patient were reviewed retrospectively, for the entire duration of the study, by experienced and board-certified radiologists. X-ray severity scores were determined in COVID-19 patients at each sampling time point according to a previously published scoring method [27]. In brief, each lung was divided into three segments: above the upper margin of the aortic arch, between the aortic arch and the lower margin of the left pulmonary artery, and below the left pulmonary artery. The extent of COVID-19 typical alterations was assessed for each segment (0 = normal lung parenchyma, 1 = only interstitial opacity, 2 = consolidation of less than 50% of the lung parenchyma, 3 = consolidation of 50% or more). A score between 0 and 18 was assigned for each patient after all six segments were analyzed for each patient.

### 2.4. Complement Assays

#### 2.4.1. Complement Protein C5a ELISA

Serum and tracheal fluid C5a was measured using a custom sandwich enzyme-linked immunosorbent assay (ELISA). Monoclonal anti-human C5a antibody (Hycult Biotech, Uden, The Netherlands) was coated on 96-well, medium-binding microplates (Greiner Bio-One, St. Gallen, Switzerland). Bound C5a was detected using a biotinylated monoclonal anti-human C5a antibody [28] and developed using avidin-alkaline phosphatase (MilliporeSigma, Merck KGaA, Darmstadt, Germany). The optical density signal was acquired using the Bio-Rad Model 680 Microplate Reader and quantified using the Microplate Manager 5.2.1 software (Bio-Rad, Hercules, CA, USA). Zymosan-induced activation of normal human serum was used as standard.

#### 2.4.2. Complement Protein Terminal Complement Complex (TCC) ELISA

Serum and tracheal fluid TCC was measured using a custom sandwich ELISA. Monoclonal anti-human C9 neoantigen antibody (Hycult Biotech) was coated on 96-well, medium-binding microplates (Greiner Bio-One). Bound TCC was detected using a custom biotinylated polyclonal anti-human C7 antibody [29] and developed using avidin-alkaline phosphatase (MilliporeSigma, Merck KGaA). The optical density signal was acquired using the Bio-Rad Model 680 Microplate Reader and quantified using the Microplate Manager 5.2.1 software (Bio-Rad). Zymosan-induced activation of normal human serum was used as standard.

### 2.5. NETosis Assay

Serum NETosis was measured using a custom sandwich ELISA. Polyclonal anti-myeloperoxidase (MPO) antibody (Invitrogen GmbH, Lofer, Austria) was coated on 96-well, high-binding microplates (Greiner Bio-One). Bound neutrophil-derived DNA was detected using a horseradish peroxidase (HRP)-conjugated anti-DNA antibody (Roche Diagnostics, Basel, Switzerland) and developed using a tetramethylbenzidine (TMB) substrate solution. The enzymatic reaction was stopped by adding 1:1 (*v*/*v*) 1M H_2_SO_4_. The optical density signal was acquired using the Bio-Rad Model 680 Microplate Reader and quantified using the Microplate Manager 5.2.1 software (Bio-Rad). Supernatant from PMA-stimulated and isolated granulocytes with defined concentration was used as standard. Briefly, granulocytes were isolated from EDTA blood using histoplaque density gradient centrifugation (350× *g*, 40 min, 18 °C). To induce NETosis, granulocytes were stimulated by adding 10µg/mL PMA to 1 × 10^7^ cells and incubating them for 4 h at 20 °C followed by centrifugation (350× *g*, 10 min, 22 °C). Supernatant was collected and stored at −20 °C until further use.

### 2.6. Viral Load and Secondary Viral Infections Analysis

SARS-CoV-2 viral load and secondary viral infections were determined by standard routine protocol at our virology department according to their respective standard operating procedures (SOPs). In short, total nucleic acid (TNA), including viral RNA or DNA, was extracted from tracheal fluid and purified using the Nuclisens EasyMag 2.0 Kit (bioMérieux, Vienna, Austria). Detection and quantification of SARS-CoV-2 viral load was achieved using the RealStar^®^ SARS-CoV-2 RT-PCR Kit 1.0 (altona Diagnostics GmbH, Hamburg, Germany). Secondary viral infections were detected by multiplex qPCR using the Fast Track Diagnostics respiratory pathogens 21 assay (Siemens Healthcare GmbH, Erlagen, Germany). PCR was performed using a CFX96 Touch real-time PCR detection system and was analyzed both manually and using CFX Manager™ Software (both from Bio-Rad). All kit-related experiments were performed according to the manufacturer’s instructions.

### 2.7. Secondary Bacterial Infections Analysis

Secondary bacterial infections were determined by standard routine protocol at our institute according to our SOPs. In short, tracheal fluid was cultured overnight on selective medium agar plates (Columbia II blood agar, cooked blood agar, MacConkey agar) at 37 °C (MacConkey agar) or 37 °C under 5% CO_2_ atmosphere (both blood agars). Species identification was achieved using matrix-laser desorption/ionization time of flight mass spectrometry (MALDI-TOF MS, Bruker Daltonik, Bremen, Germany) combined with the reference Biotyper library v4.1 (Bruker Daltonik).

### 2.8. Hemoglobin Assay

Quantification of blood in tracheal fluid samples was performed using a Hemoglobin Assay Kit (Sigma-Aldrich, Merck KGaA). Samples with a value of ≥200 mg/dL were classified to be high in blood content levels and assumed that tracheal fluid was contaminated with blood during suction procedure. Thus, these samples were excluded from further local infections studies.

### 2.9. Statistical Analysis

Continuous parameters are presented as mean ± standard deviation (SD) and nominal data is shown as numbers with percentage. Differences between patient and control groups were analyzed using unpaired *t*-test or Mann–Whitney U test for continuous parameters based on normal distribution testing and Fisher’s exact test or chi-square test for categorical parameters. Kinetic studies of continuous parameters comparing different subgroups was approached by linear mixed model of log-transformed data. Correlation studies between continuous variables were assessed using the Spearman rank correlation test. *p* < 0.05 (2-tailed) was considered a statistically significant difference. Bonferroni correction was applied were multiple testing was applicable.

## 3. Results

### 3.1. Patient Characteristics

**Figure 1:**
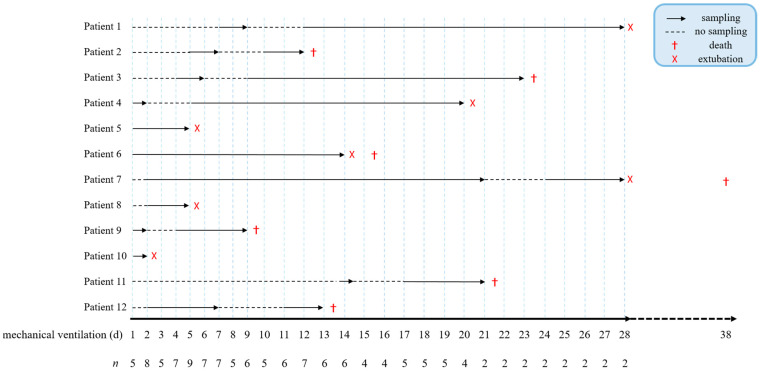
Sampling timeline of COVID-19 patients. Continuous sampling of serum and tracheal fluid began on the day that mechanical ventilation was required, and *n* refers to the number of patients from whom samples were received per day. Arrows indicate sampling, dotted lines represent sampling breaks, (X) marks patient extubation, and (†) the death of patients.

A total of 12 patients and 13 healthy controls were recruited for this study. SARS-CoV-2 patients had a mean age of 79.2 ± 5.1 years, were predominantly males (83.3%), and required mechanical ventilation for 14.8 ± 9.1 days. Seven patients (58.3%) died during mechanical ventilation (*n* = 5) or within 10 days after the end of artificial respiration (*n* = 2) (Figure 1 and Table 1).

The control group exhibited significant differences in age, gender and mortality. On average, the first patient samples were available on day 2 (1–5) of mechanical ventilation (data shown as median (IQR)). Baseline values of common systemic inflammation, but not coagulation markers in blood, were significantly elevated in patients compared to healthy controls (Table 1).

Complement activation markers C5a and TCC were significantly elevated in patient baseline sera compared to healthy controls with values of 24.5 ± 39.0 ng/mL and 11.03 ± 8.52 µg/mL and a significance of *p* = 0.0037 and *p* < 0.0001, respectively. In addition, the release of systemic NETs was significantly higher in patient samples compared to the control group with levels of 5.87 (±3.71) × 10^6^ neutrophils/mL vs. 0.82 (±0.74) × 10^6^ neutrophils/mL (*p* < 0.0001; Table 1).

### 3.2. Viral Load and Secondary Infections

Viral load in tracheal fluid was obtained by routinely performed RT-qPCR. Strikingly, one patient did not show a positive PCR result despite being hospitalized for COVID-19. However, the remaining patients that survived or died after assisted ventilation showed a mean Ct value of 27.2 ± 2.7 or 26.2 ± 3.3, respectively. There was no difference observed in Ct values between survivors and nonsurvivors over time (Figure S1a). In total, four (two survivors and two nonsurvivors) out of 12 COVID-19 patients with PCR confirmed SARS-CoV-2 infections presented negative PCR results during the course of the disease. Individuals from the control group were all negative for SARS-CoV-2.

Secondary respiratory infections were determined by our bacterial or viral routine laboratory SOP, as described in Section 2.6 or Section 2.7. No viral infections were present in our patients; however, secondary bacterial infections with yeast, *Escherichia coli,* and/or *Aspergillus fumigatus* were detected (*n* = 7). In tracheal fluid of healthy donors, non-pathogenic traces of Enterovirus, *Influenza*, Rhinovirus, *Moraxella catarrhalis*, and/or yeast were detected in three individuals (Table S1).

### 3.3. Elevated Systemic Inflamatory Processes and Activation of Complement but No Increased NETs Release in Nonsurvivors

**Figure 2:**
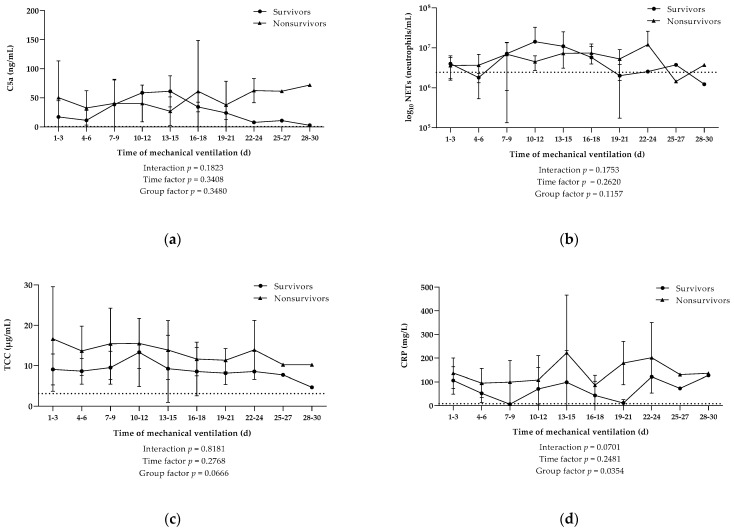
Kinetics of systemic activation of different parameters in blood. Levels of (**a**) complement protein C5a, (**b**) neutrophilic extracellular traps (NETs), (**c**) terminal complement complex (TCC), and (**d**) CRP were measured during the course of mechanical ventilation of COVID-19 patients that survived (*n* = 5) or died (*n* = 7). Data are shown as mean ± SD. Dotted lines indicate the mean value in the control group. *p*-Values were calculated by linear mixed model of clustered log-transformed data.

No significant difference in baseline values of serum C5a, TCC, and NETosis was found between survivors and nonsurvivors with ranging levels of 15.06 ± 25.56 ng/mL vs. 31.17 ± 47.13 ng/mL, 8.49 ± 3.18 µg/mL vs. 12.85 ± 10.82 µg/mL, and 6.58 (±3.81) × 10^6^ neutrophils/mL vs. 5.36 (±3.85) × 10^6^ neutrophils/mL, respectively. The initial results of the kinetic studies confirmed similar levels of serum C5a as well as comparable concentrations of neutrophils that released NETs in the bloodstream of survivors and nonsurvivors over the period of mechanical ventilation (Figure 2a,b). The levels of systemic TCC in our cohort consisting of 12 patients were overall higher in nonsurvivors compared to survivors over the course of mechanical ventilation (Figure 2c).

Kinetic studies of single laboratory parameters revealed significantly elevated CRP levels in nonsurvivors, when compared to survivors, during artificial respiration (*p* = 0.0354; Figure 2d). However, other investigated inflammation and coagulation markers did not reveal any differences between patients that survived and those that died due to severe SARS-CoV-2 infection (Figure S2).

The correlation between complement and NETosis parameters in sera was determined using the Spearman rank correlation test. C5a and TCC showed a strong significant positive correlation (*r* = 0.5417, *p* < 0.0001; Figure S3a). Contrarily, correlation of NETs release with the complement activation products C5a and TCC merely revealed weak positive and negative trends, respectively (Figure S3b,c).

### 3.4. Systemic Complement Activation and NETosis Significantly Associated with Inflammatory and Coagulation Markers in COVID-19 Patients

**Figure 3:**
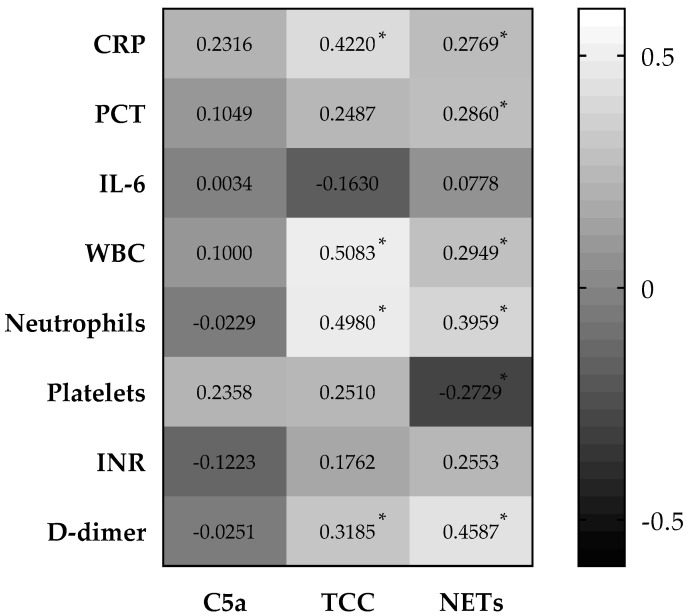
Correlation matrix of activation in systemic parameters. Complement activation products, C5a and TCC, and NETs release correlated with common inflammation and coagulation markers in mechanically ventilated patients with SARS-CoV-2 infection. Correlation coefficient was assessed using the Spearman rank correlation test. Values indicate Spearman r. CRP, C-reactive protein; PCT, procalcitonin; IL-6, interleukin 6; WBC, white blood cell count; INR, international normalized rate; TCC, terminal complement complex; NETs, neutrophilic extracellular traps. * *p* < 0.002 (significant after Bonferroni correction).

Upon assessing the association of systemic complement activation and the release of NETs with common inflammation (CRP, PCT, IL-6, WBC, and neutrophil count) and coagulation (platelets, INR, D-dimer) markers over the period of mechanical ventilation, we observed multiple correlations (Figure 3 and Figures S4 and S5). Whereas C5a showed no significant correlation with any of the investigated laboratory parameters, TCC presented a significant positive correlation with CRP, WBC count, neutrophil count, and D-dimer. The highest number of significant correlations was observed in NETs release. This parameter showed a significant positive correlation with CRP, PCT, WBC count, neutrophil count, and D-dimer, whereas platelet count displayed a significant negative correlation.

### 3.5. Detection of Local Complement Activation and NETosis in Tracheal Fluid throughout Mechanical Ventilation

Hemoglobin assay identified 16 out of 132 tracheal fluid samples from SARS-CoV-2 patients with high levels of hemoglobin values (≥200 mg/dL). None of the samples from the control group had high levels of hemoglobin. Consequently, samples with high hemoglobin content were excluded from complement and NETosis analysis. Hereby, one survivor (patient 10) had to be excluded completely. Of the remaining patient samples (*n* = 116), no significant discrepancy in hemoglobin content was observed between survivors and nonsurvivors at baseline values or over the time of mechanical ventilation (Figure S1b).

Initial TCC levels in tracheal fluid significantly differed between patients and healthy controls, with concentrations detected at 0.81 ± 0.59 µg/mL and 0.26 ± 0.23 µg/mL (*p* = 0.0051), respectively. Baseline values of C5a, NETs release, and hemoglobin in tracheal fluid tended to be elevated in patients; nevertheless, they exhibited no significant difference compared to the control group (Table 2).

**Figure 4:**
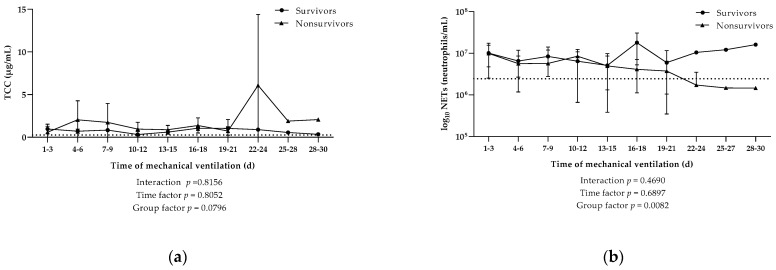
Kinetics of local activation of different parameters in tracheal fluid. Kinetics of local (**a**) complement activation characterized by the terminal complement complex (TCC) and (**b**) the release of NETs from neutrophils measured during the course of mechanical ventilation in COVID-19 patients that survived (*n* = 4) or died (*n* = 7). Data are shown as mean ± SD. Dotted lines indicate the mean value in the control group. *p*-Values were calculated by linear mixed model of clustered log-transformed data.

In line with the results obtained from sera, no initial differences were found in C5a, TCC, and NETosis in tracheal fluid of survivors and nonsurvivors, with levels measuring at 0.89 ± 1.07 ng/mL vs. 3.81 ± 8.98 ng/mL, 0.86 ± 0.60 µg/mL vs. 0.78 ± 0.63 µg/mL, and 14.02 (±14.20) × 10^6^ neutrophils/mL vs. 65.50 (±63.19) × 10^6^ neutrophils/mL, respectively. In general, C5a was present in tracheal fluid but in very little amounts; thus, no kinetic studies were performed for this anaphylatoxin. TCC levels in tracheal fluid revealed no significant difference between survivors and non survivors (Figure 4a). In contrast, the concentration of neutrophils undergoing NETosis was significantly higher in survivors over the course of mechanical ventilation (*p* = 0.0082; Figure 4b).

Correlation analysis of TCC and NETs release exhibited a weak negative trend (*p* = −0.1875; Figure S6a). Moreover, there was no correlation observed between the viral load and the levels of TCC or NETs in the tracheal fluid of COVID-19 patients (Figure S6b,c).

### 3.6. Evalution of LungTissue Based on Chest X-ray Severity Score and Implementation of a Severity Ratio

**Figure 5:**
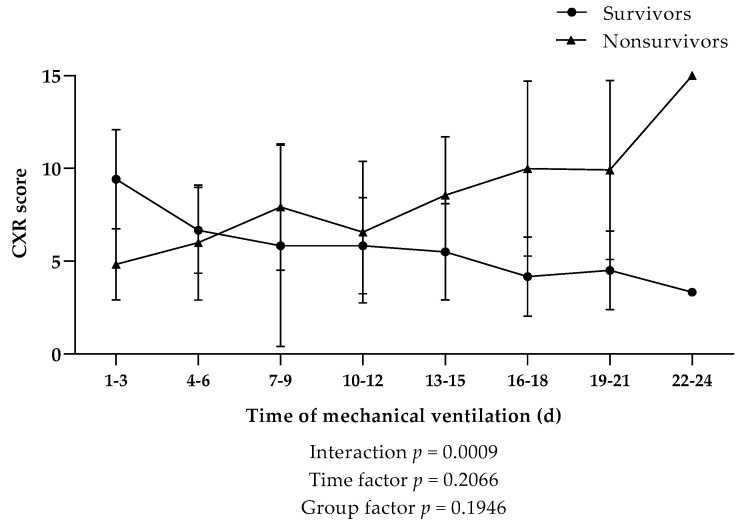
Kinetics of the chest X-ray (CXR) score during the course of mechanical ventilation in COVID-19 patients that survived (*n* = 4) or died (*n* = 7). Data are shown as mean ± SD. Dotted line indicates the survival threshold. *p*-Values were calculated by linear mixed model of clustered log-transformed data.

CXR analysis and scoring was used to determine disease severity in the lung tissue of our patient cohort. A general trend revealed a higher CXR score in nonsurvivors; however, no significant difference in CXR scores was observed between nonsurvivors and survivors over the time of mechanical ventilation (Figure 5).

**Figure 6:**
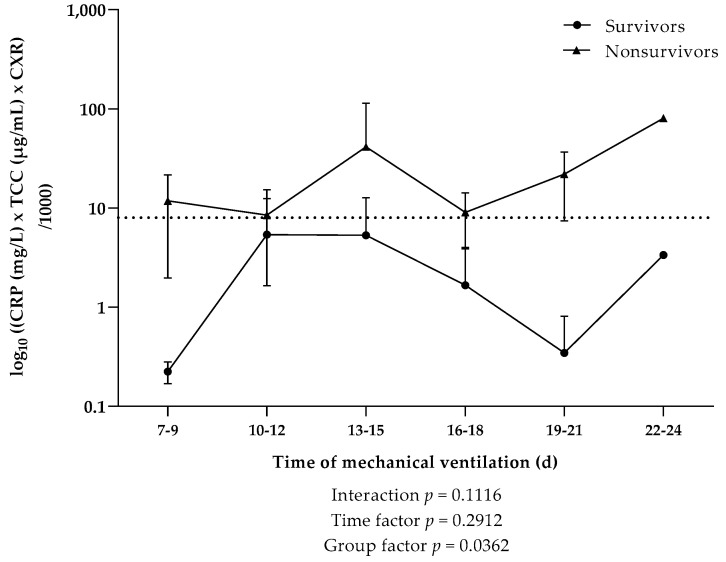
Kinetics of a ratio consisting of systemic C-reactive protein (CRP), terminal complement complex (TCC), and CXR score during the course of mechanical ventilation in COVID-19 patients that survived (*n* = 4) or died (*n* = 7). Data are shown as mean ± SD. Dotted line indicates the survival threshold. *p*-Values were calculated by linear mixed model of clustered log-transformed data.

In order to pronouncedly indicate the interplay of the three specific parameters (CRP, TCC, and CXR score), which independently displayed overall elevated levels in nonsurvivors during the course of mechanical ventilation, a ratio of CRP × TCC × CXR kinetics was introduced (Figure 6). CXR scores in the two patient cohorts revealed a significant interaction in the first week of mechanical ventilation (Figure 5); therefore, values from days 1–6 were omitted from our analysis. Starting from the second week of mechanical ventilation, nonsurvivors showed a significantly higher CRP × TCC × CXR ratio compared to survivors. We further observed, in this specific cohort, that the mean values of CRP × TCC × CXR were consistently above 8 in nonsurvivors, while values in survivors did not exceed 5.4.

No significant correlation was observed between CXR scores and the complement activation products C5a (Figure S7a), serum TCC (Figure S7b), and tracheal fluid TCC (Figure S7c). Similarly, CXR scores displayed no significant correlation with NETs release in serum (Figure S7d) or tracheal fluid (Figure S7e).

## 4. Discussion

The complement system triggers the production of anaphylatoxins (C3a and C5a) and the formation of the terminal complement complex and is known to interact with the coagulation cascade, thereby instigating neutrophil activation and NETosis. Henceforth, an increase in complement activation, signaled by elevated levels of complement activation products, has been heavily implicated in severe COVID-19 [17,30].

In our longitudinal study, we observed significantly higher baseline levels of C5a and TCC in sera of COVID-19 patients compared to the control group. These results have been reflected in a study where higher baseline levels of TCC were reported in COVID-19 patients, albeit C5a levels remained under the detection limit [24]. Several other studies have showcased an increase in systemic TCC [17,31] and C5 or C5a [18,19] levels in COVID-19 patients compared to healthy donors. The systemic overactivation of the complement system may eventually indicate the outcome of disease in severe COVID-19 cases. Studies even identified the activation of the complement system via alternative and MBL pathway as a mortality indicator [32,33].

The unique inflammatory response exhibited by neutrophils whereby they release NETs as an effector mechanism is associated with poor clinical outcome in COVID-19 [34]. Several studies revealed the role of neutrophils in the pathology of the disease as levels of blood neutrophils and NETs released in COVID-19 patients were higher than those in a healthy cohort [35,36]. We have similarly noted higher levels of NETs release in sera of COVID-19 patients and further highlighted a strong correlation between NETs and inflammatory and coagulation markers, mainly CRP, PCT, WBC count, neutrophil count, and D-dimer. Indeed, NETs release has been associated with several clinical biomarkers, including acute-phase reactants (CRP and D-dimer) and inflammatory cytokines [37,38], in COVID-19 patients and is rendered a prognostic marker for diseases severity [39]. The activation of neutrophils and the formation of NETs could therefore be a potential therapeutic target for critical COVID-19 cases.

Kinetic evaluation of systemic C5a, TCC, and NETosis revealed significantly higher values of TCC in nonsurvivors, whereas C5a and NETosis levels did not differ between survivors and nonsurvivors. TCC, a terminal product of complement activation, represents a stable complex that remains in fluid phase, whereas the anaphylatoxin C5a is bound by receptors upon cleavage of C5 and is thus a less stable marker of complement activation. Significantly higher TCC levels have been previously reported in ICU patients when compared to non-ICU patients [40]. In a longitudinal cohort of COVID-19 patients, TCC levels were consistently elevated in patients under mechanical ventilation and significantly higher compared to asymptomatic patients or those with mild symptoms [41]. The heightened activation of complement components can evidently be rendered as critical indicators of disease severity.

Our investigation of local kinetics in tracheal fluid yielded no significant differences between survivors and nonsurvivors in regards to C5a and TCC activation. We detected minute amounts of C5a in the tracheal fluid, which could be explained by the increased local activation of neutrophils in the lungs. It may be that the crosstalk between C5a and C5a receptor (C5aR) expressed on neutrophils drove towards the consumption of local C5a in the lungs, making it difficult to detect C5a locally.

It is worth noting that one of the most consistent observations reported across COVID-19 clinical studies, including our study, is patient characteristics. Patients most frequently admitted to hospital care, especially those in need of mechanical ventilation, were generally 65+ year-old males, a demographic that has been a hallmark of critical COVID-19 [42]. Several factors have been considered as potential predictors for the requirement of mechanical ventilation, the most prominent being IL-6 and CRP [43,44]. Levels of IL-6 and CRP have been shown to increase proportionally to disease severity [41,45]. CRP levels in our patient cohort were elevated compared to control, and we further observed significantly higher CRP levels in nonsurvivors compared to survivors. Interestingly, IL-6 and CRP were assessed as predictive values for respiratory deterioration, where CRP levels above 97 mg/L indicated the need for mechanical ventilation [43]. Although the average CRP levels in our patient cohort was 111 mg/L, we cannot infer from our small sample size that CRP levels are correlated with our cohort’s survival or need of mechanical ventilation. Nevertheless, the careful monitoring of these parameters could facilitate early identification of COVID-19 patients at risk of respiratory failure.

High-resolution computed tomography (HR-CT) is deemed the gold standard for the radiologic evaluation of pulmonary alteration severity in COVID-19 infections. HR-CT is highly sensitive at detecting lung alterations that are typical in COVID-19-pneumonia, including ground glass opacity, crazy paving, bronchiectasis, and consolidation. Furthermore, HR-CT can differentiate, with high specificity, between these COVID-19-typical alterations and other causes of lung opacity, like pleural effusion, atelectasis, or bacterial pneumonia.

CT is more specific and sensitive at detecting typical COVID-19 lung alterations compared to conventional chest X-ray (CXR). Nevertheless, CXR remains a useful tool for assessing the progression of infection in patients with known COVID-19-pneumonia. Several studies comparing severity scores accuracy, based on CXR and CT analysis, revealed low interobserver variability and positive correlation between CXR- and CT-based scores, with a slight overestimation of CXR-scores compared to CT [46].

The assessment of several parameters revealed significantly higher values of a CRP × TCC × CXR ratio in nonsurvivors compared to survivors. It is worth noting that the mean values of the CRP × TCC × CXR ratio did not drastically differ from that of the CRP × TCC ratio (Figure S8); however, the inclusion of the CXR score was significant, as it allowed us to pinpoint a value that distinguished between survivors and nonsurvivors. In our cohort, we specifically showcased that nonsurvivors had a mean value above 8 throughout the period of mechanical ventilation, indicating that COVID-19 patients above this value may be at a critical, fatal state. Evidently, our sample size is too small, and a larger cohort would provide a better intel on the actual role of CXR as a severity score in COVID-19. Consequently, with a larger cohort, one could confirm our hypothesis that a higher CRP × TCC × CXR ratio could serve as a predictive parameter of disease severity and survivability.

In conclusion, our study confirms the critical role of complement activation, systemically and locally, in SARS-CoV-2 infections. Furthermore, we could demonstrate that local NETosis in the lungs is a predictive parameter of mortality in patients under mechanical ventilation. Our results suggest that a combination of high systemic TCC levels and decreased local NETosis can strengthen the prediction of infection severity. Lastly, we propose a specific cutoff value of a ratio consisting of systemic CRP and TCC as well as CXR score as a discriminating factor of survivability in COVID-19 patients. The proposed ratio is uninformative at the time of admission; however, it could later serve as a measure in the timeline of therapeutic intervention. Of course, our results herein need to be confirmed by extended studies.

## Figures and Tables

**Table 1:** Characteristics as well as systemic and experimental laboratory blood parameters of patients and healthy controls. Values represent baseline parameters acquired at first sampling time point. Data are presented as mean ± SD or *n* (percentage). Differences between both groups were analyzed by unpaired *t*-test, Mann–Whitney U test, or Fisher’s exact test, as appropriate.

	SARS-CoV-2 Patients	Healthy Controls	*p*-Value
Epidemiological Parameters			
*n*	12	13	n.a.
Age (years)	79.2 ± 5.1	62.4 ± 11.5	***
Gender *n* f/m (%)	2/10 (16.7/83.3)	8/5 (61.5/38.5)	*
Mechanical ventilation (days)	14.8 ± 9.1	n.a.	n.a.
Mortality *n* (%)	7 (58.3)	0 (0.0)	**
Laboratory Parameters			
CRP (mg/L)	111.0 ± 67.1	8.2 ± 9.0	****
PCT (ng/mL)	1.8 ± 4.1	n.a.	n.a.
IL-6 (pg/mL)	3009 ± 9694	n.a.	n.a.
WBC (cells/µL)	12,388 ± 3993	8838 ± 3061	*
Neutrophils (cells/µL)	10,812 ± 4026	n.a.	n.a.
Platelets (cells/µL)	296,583 ± 150,973	291,658 ± 74,335	ns
INR	1.2 ± 0.3	1.1 ± 0.1	ns
D-Dimer (mg/L)	3.5 ± 3.0	n.a.	n.a.
Experimental Parameters			
C5a (ng/mL)	24.5 ± 39.0	0.8 ± 0.5	**
TCC (µg/mL)	11.03 ± 8.52	3.14 ± 1.89	****
NETs (neutrophils/mL)	5.87 (±3.71) × 10^6^	0.82 (±0.74) × 10^6^	****

n.a., not applicable; CRP, C-reactive protein; PCT, procalcitonin; IL-6, interleukin 6; WBC, white blood cell count; INR, international normalized rate; TCC, terminal complement complex; NETs, neutrophilic extracellular traps; ns, not significant. * *p* < 0.05, ** *p* < 0.01, *** *p* < 0.001, **** *p* < 0.0001.

**Table 2:** Complement, NETosis, and hemoglobin levels in tracheal fluid of patients and healthy controls. Values represent baseline levels at first sampling time point. Data are presented as mean ± SD or *n* (percentage). Differences between both groups were analyzed by unpaired *t*-test or Mann–Whitney U test, as appropriate.

Experimental Parameters	SARS-CoV-2 Patients	Healthy Controls	*p*-Value
C5a (ng/mL)	2.7 ± 7.1	0.3 ± 0.4	ns
TCC (µg/mL)	0.81 ± 0.59	0.26 ± 0.23	**
NETs (neutrophils/mL)	9.54 (±10.2) × 10^6^	2.45 (±1.58) × 10^6^	ns
Hb (mg/dL)	45.0 ± 41.8	24.2 ± 17.5	ns

TCC, terminal complement complex; NETs, neutrophilic extracellular traps; Hb, hemoglobin. ns: not significant; ** *p* < 0.01.

## Supplementary Materials

The following are available online at https://www.mdpi.com/article/10.3390/v13122376/s1, Table S1: List of bacterial and viral secondary respiratory infections in patients and healthy controls; Figure S1: Kinetics of Ct values and hemoglobin in tracheal fluid; Figure S2: Kinetics of common systemic inflammation and coagulation markers; Figure S3: Correlation between systemic complement activation products and NETosis; Figure S4: Correlation of serum complement activation products and NETs release with common inflammation markers; Figure S5: Correlation of serum complement activation products and NETs release with common coagulation markers; Figure S6: Correlation between complement activation products and NETosis in tracheal fluid of COVID-19 patients; Figure S7: Correlation between chest X-ray (CXR) scores and complement activation products or NETosis in COVID-19 patients; Figure S8: Kinetics of a ratio of systemic inflammation and complement activation marker.
