# Supplementary material for: Systemic Inflammation and Complement Activation Parameters Predict Clinical Outcome of Severe SARS-CoV-2 Infections
Source: Viruses. 2021 Nov 26;13(12):2376. doi: 10.3390/v13122376 (PMC8707937; doi:10.3390/v13122376)
Supplement: Supplementary file 1 [file viruses-13-02376-s001.zip › viruses-1450029-supplementary.pdf]

Viruses

Systemic Inflammation and Complement Activation Parameters Predict Clinical Outcome of Severe SARS-CoV-2 Infections

Supplementary Materials

Silke Huber [1, †], Mariam Massri [1, †], Marco Grasse [1], Verena Fleischer [1], Sára Kellnerová [1], Verena Harpf [1], Ludwig Knabl [2], Ludwig Knabl Sr. [3], Tatjana Heiner [4], Moritz Kummann [5], Magdalena Neurauter [1], Günter Rambach [1], Cornelia Speth [1] and Reinhard Würzner [1,*]

[*] Corresponding author

[†] These authors contributed equally to this work

[1]Institute of Hygiene and Medical Microbiology, Medical University of Innsbruck, Innsbruck, Austria

[2]Pathology Laboratory, 6511 Zams, Austria

[3]Department of Internal Medicine, Hospital St. Vinzenz, 6511 Zams, Austria

[4]Department of Anesthesia and Intensive Care Medicine, Hospital Reutte, 6600 Reutte, Austria

[5]Department of Radiology. Medical University of Innsbruck, 6020 Innsbruck, Austria

Correspondence to:

Ao. Univ.-Prof. Dr.med. Reinhard Würzner, PhD

Institute of Hygiene and Medical Microbiology

Medical University of Innsbruck

Schöpfstrasse 41

6020 Innsbruck, Austria

Email: reinhard.wuerzner@i-med.ac.at

Phone: +43 (0)512 9003 70707

Table S1. List of bacterial and viral secondary respiratory infections in patients and healthy controls. Individuals not represented in this table had negative results for both analyses.

	Bacterial Infections	Viral Infections
**Patients**		
Patient 1	yeast, *Escherichia coli*	negative
Patient 3	*Aspergillus fumigatus* complex	negative
Patient 6	yeast	negative
Patient 7	*Escherichia coli*	negative
Patient 8	yeast	negative
Patient 9	*Aspergillus fumigatus* complex	negative
Patient 11	yeast	negative
**Healthy controls**		
Healthy Control 6	negative	Enterovirus, *Influenza* (low)
Healthy Control 9	*Moraxella catarrhalis*	Rhinovirus
Healthy Control 13	Yeast	negative

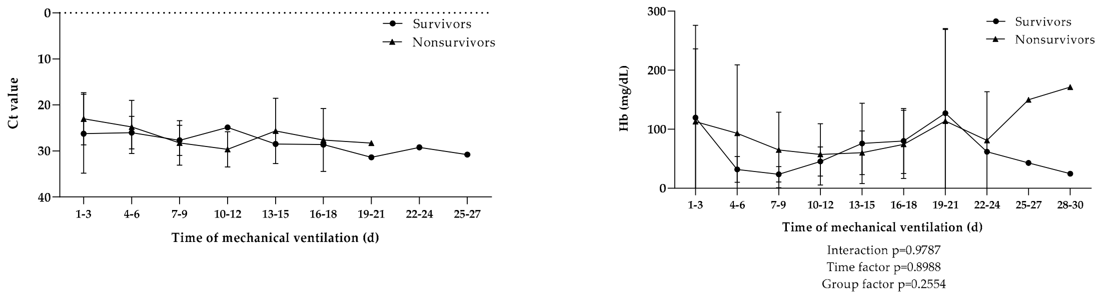

(**a**) (**b**)

Figure S1. Kinetics of Ct values and hemoglobin in tracheal fluid. (**a**) Ct values and (**b**) hemogoblin (Hb) levels in tracheal fluid of COVID-19 patients that survived (n=4) or died (n=7) during the course of mechanical ventilation. Data is shown as mean ± SD. Dotted lines indicate the mean value in the control group. P-values were calculated by linear mixed model of clustered log-transformed data.

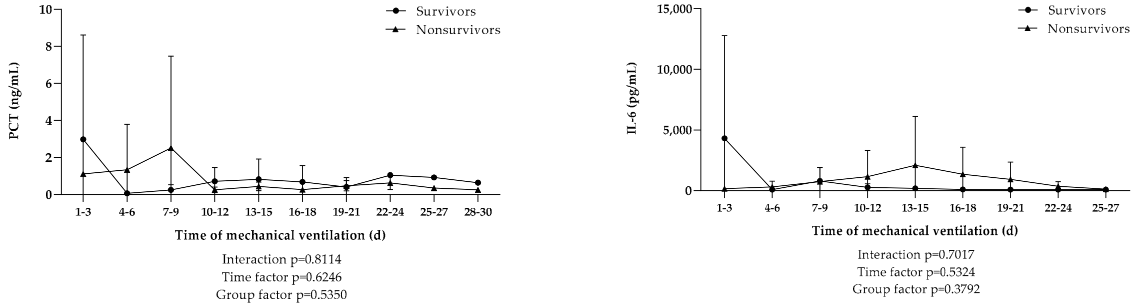

(**a**) (**b**)

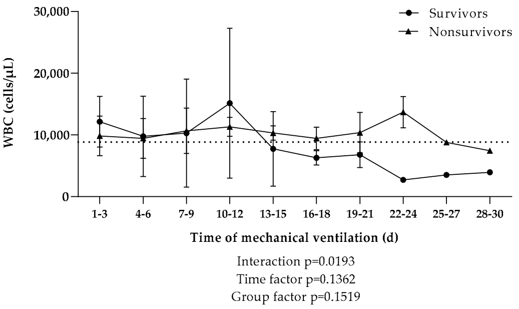

(**c**)

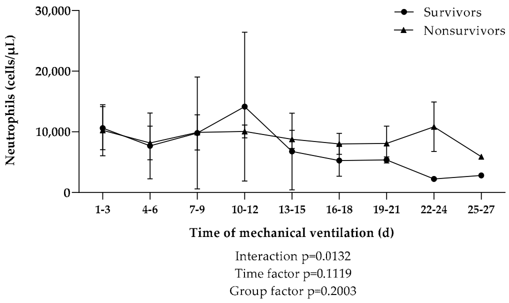

(**d**)

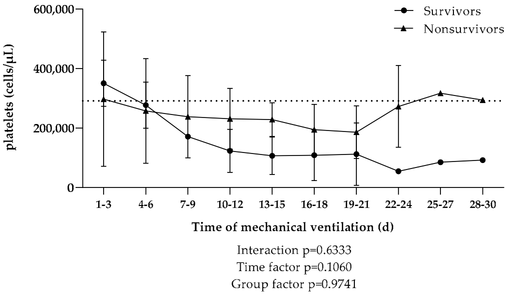

(**e**)

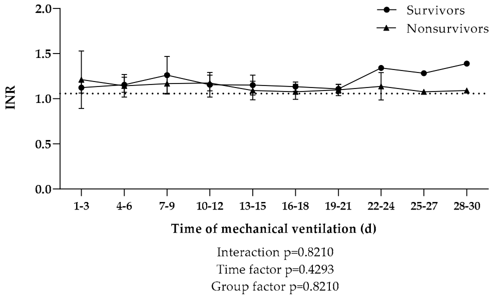

(**f**)

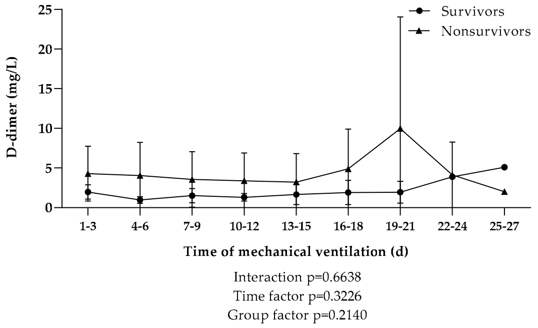

(**g**)

Figure S2. Kinetics of common systemic inflammation and coagulation markers. (a) procalcitonin (PCT), (b) interleukin-6 (IL-6), (c) white blood cell (WBC) count, (d) neutrophil count, (e) platelets count, (f) international normalized rate (INR), and (g) D-dimer during course of mechanical ventilation of COVID-19 patients that survived (n=5) or died (n=7). Data is shown as mean ± SD. Dotted lines indicate the mean value in the control group. P-values were calculated by linear mixed model of clustered log-transformed data.

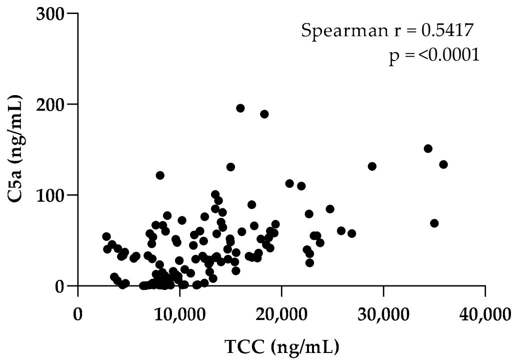

(a)

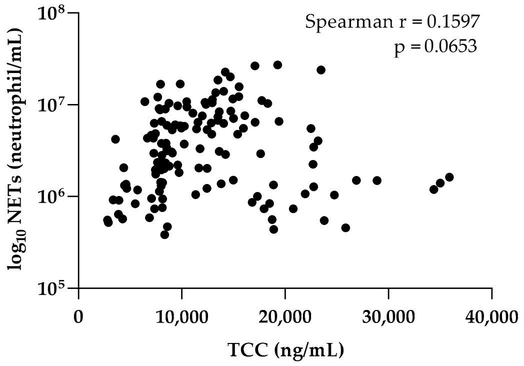

(b)

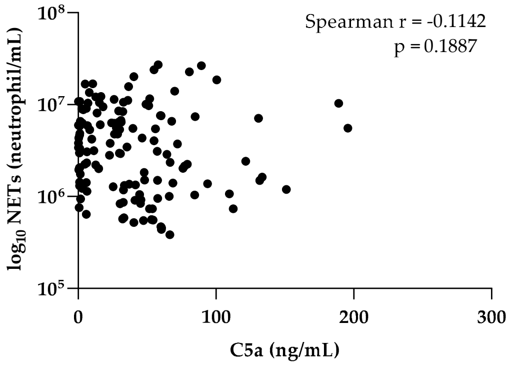

(c)

Figure S3. Correlation between systemic complement activation products and NETosis. (**a**) C5a *vs.* TCC, (**b**) NETs release *vs.* TCC, and (**c**) release of NETs *vs.* C5a. Correlation coefficient was assessed using the Spearman rank correlation test. Correlation coefficient was assessed using the Spearman rank correlation test. Values indicate Spearman r. CRP: C-reactive protein, PCT: procalcitonin, IL-6: interleukin 6, WBC: white blood cell count, INR: international normalized rate, TCC: terminal complement complex, NETs: neutrophilic extracellular traps. $p<0.02$ were considered significant after Bonferroni correction for multiple testing.

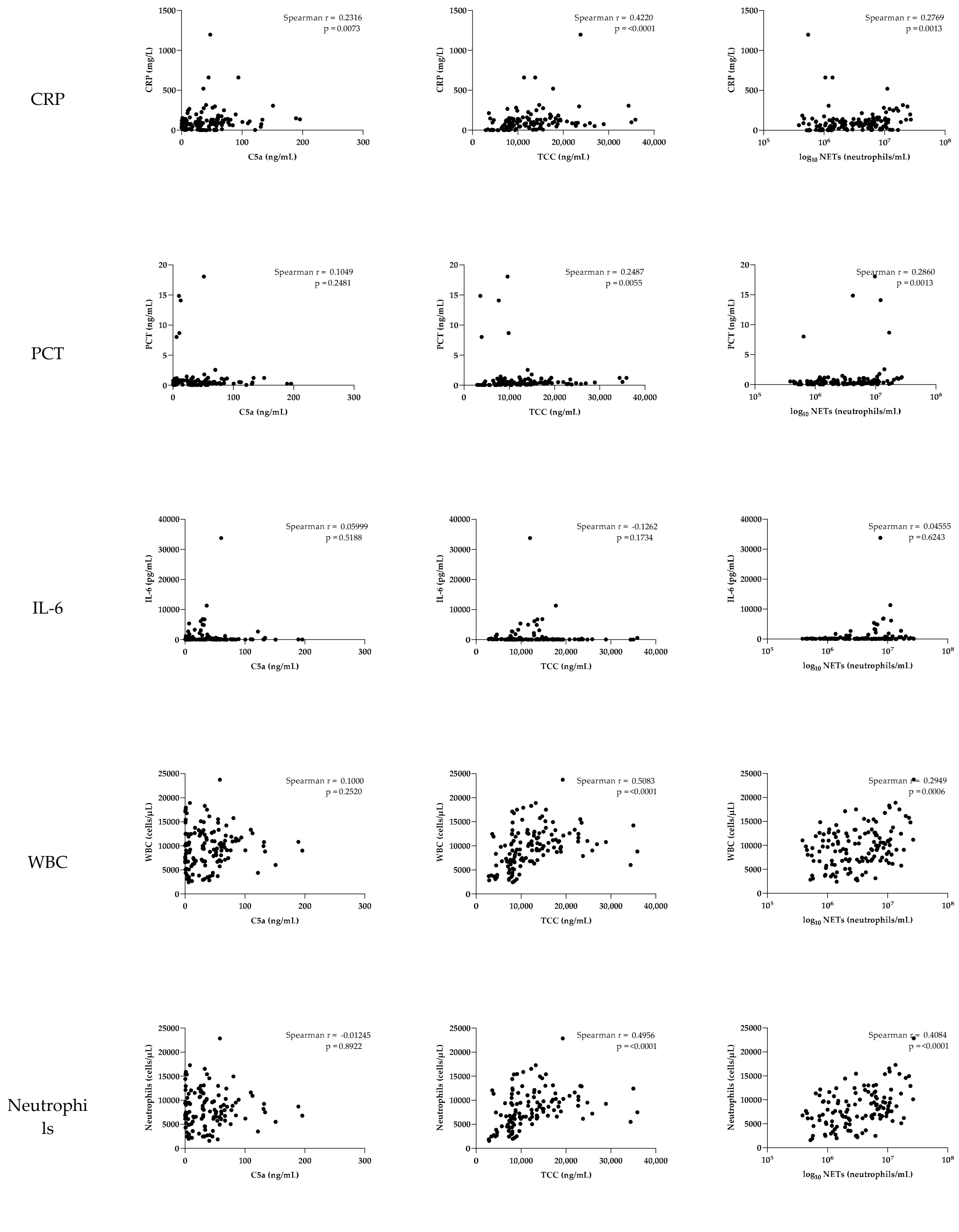

Figure S4. Correlation of serum complement activation products and NETs release with common inflammation markers. Correlation coefficient was assessed using the Spearman rank correlation test. CRP: C-reactive protein, PCT: procalcitonin, IL-6: interleukin 6, WBC: white blood cell count, INR: international normalized rate, TCC: terminal complement complex, NETs: neutrophilic extracellular traps. $p<0.002$ were considered significant after Bonferroni correction for multiple testing.

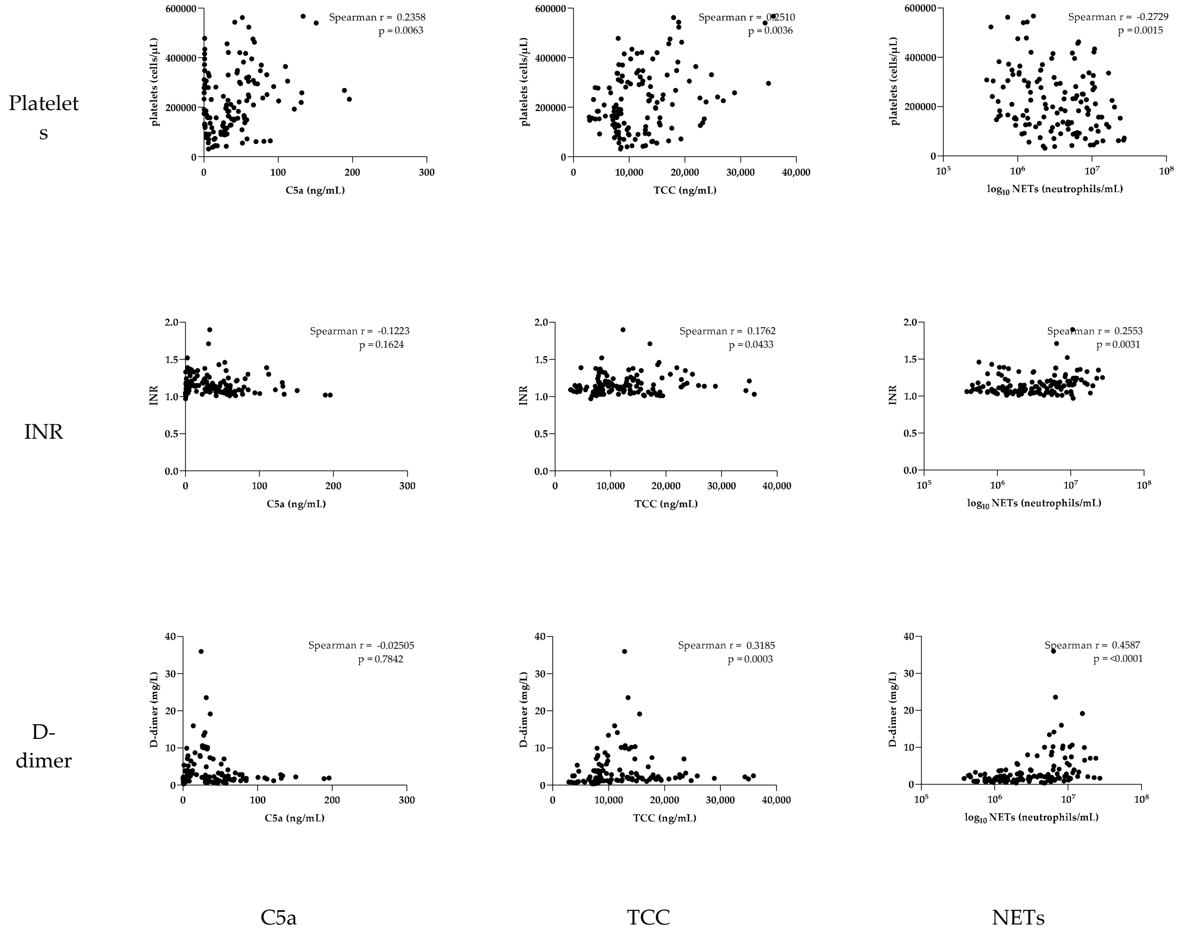

Figure S5. Correlation of serum complement activation products and NETs release with common coagulation markers. Correlation coefficient was assessed using the Spearman rank correlation test. CRP: C-reactive protein, PCT: procalcitonin, IL-6: interleukin 6, WBC: white blood cell count, INR: international normalized rate, TCC: terminal complement complex, NETs: neutrophilic extracellular traps. p<0.006 were considered significant after Bonferroni correction for multiple testing.

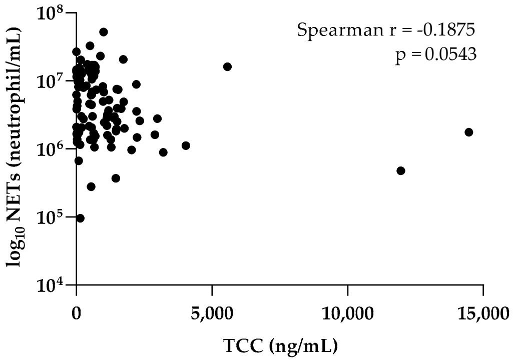

(**a**)

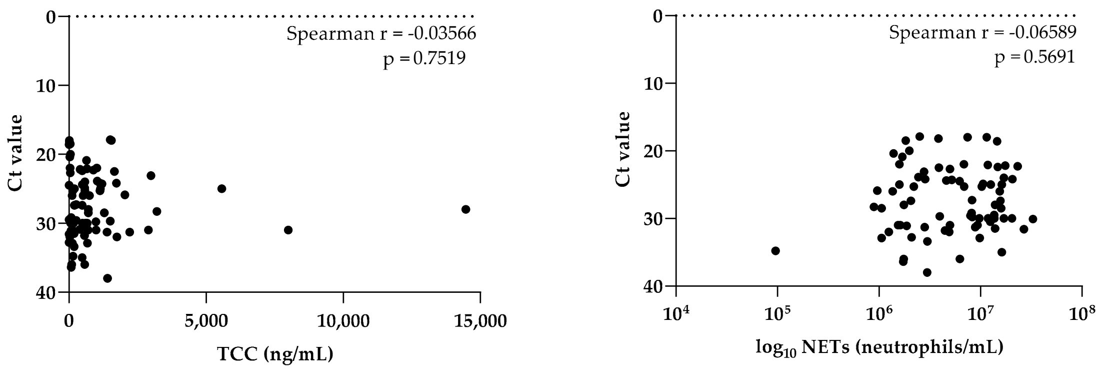

(**b**) (**c**)

Figure S6. Correlation between complement activation products and NETosis in tracheal fluid of COVID-19 patients. (**a**) C5a *vs.* terminal complement complex (TCC), (**b**) (neutrophilic extracellular traps) NETs release *vs.* TCC, and (**c**) release of NETs *vs.* C5a. Correlation coefficient was assessed using the Spearman rank correlation test. $p<0.02$ were considered significant after Bonferroni correction for multiple testing.

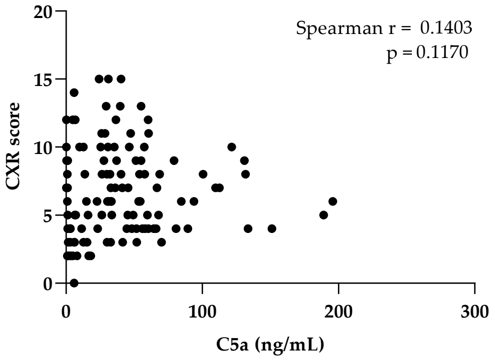

(**a**)

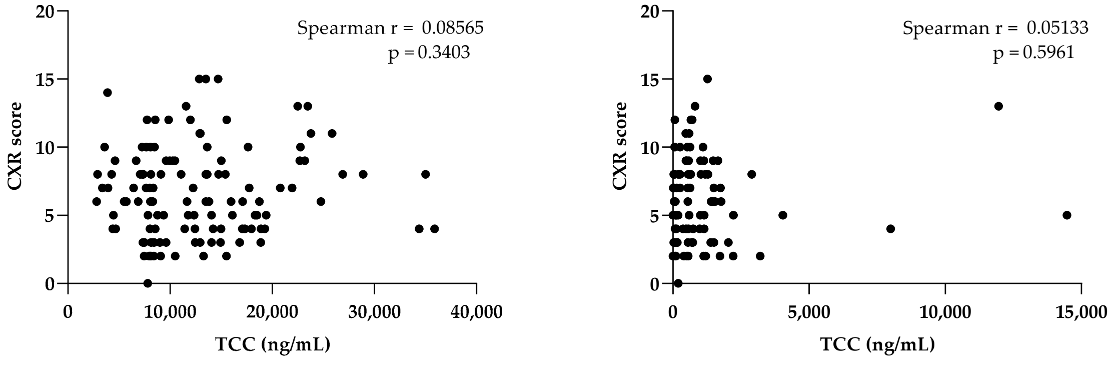

(b) (c)

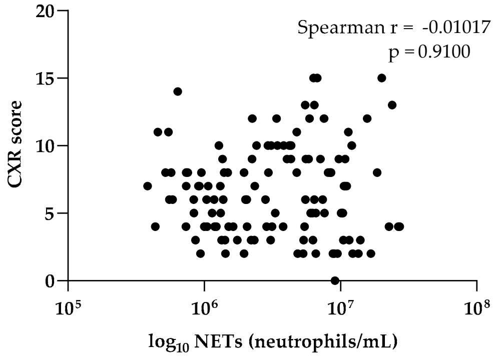

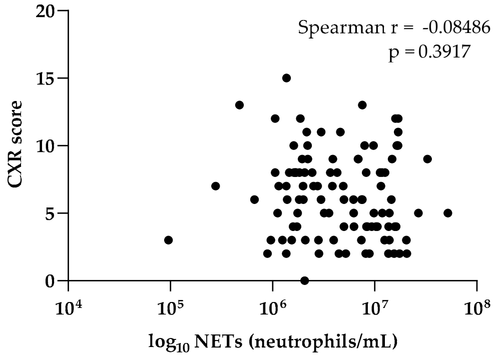

(d) (e)

Figure S7. Correlation between chest x-ray (CXR) scores and complement activation products or NETosis in COVID-19 patients. (**a**) CXR score *vs.* C5a in serum, (**b**) CXR score *vs.* terminal complement complex (TCC) in serum, (**c**) CXR score *vs.* TCC in tracheal fluid (**d**) CXR score *vs.* release of neutrophilic extracellular traps (NETs) in serum, and (**e**) CXR score *vs.* release of neutrophilic extracellular traps (NETs) in tracheal fluid. Correlation coefficient was assessed using the Spearman rank correlation test. $p<0.01$ were considered significant after Bonferroni correction for multiple testing.

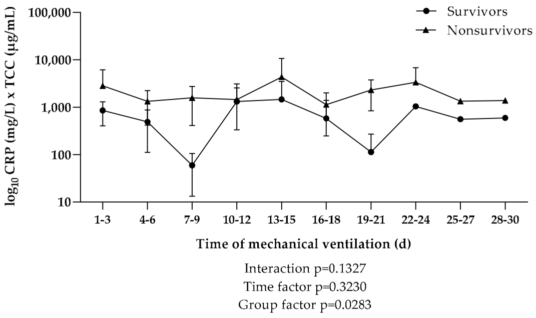

Figure S8. Kinetics of a ratio of systemic inflammation and complement activation marker. A ratio consisting systemic C-reactive protein (CRP) and terminal complement complex (TCC) levels in COVID-19 patients that survived (n=4) or died (n=7) during the course f mechanical ventilation. Data is shown as mean ± SD. P-values were calculated by linear mixed model of clustered log-transformed data.
